# Identification of 7-(4′-Cyanophenyl)indoline-1-benzenesulfonamide as a mitotic inhibitor to induce apoptotic cell death and inhibit autophagy in human colorectal cancer cells

**DOI:** 10.1038/s41598-017-12795-5

**Published:** 2017-09-29

**Authors:** Tung-Yun Wu, Ting-Yu Cho, Chung-Kuang Lu, Jing-Ping Liou, Mei-Chuan Chen

**Affiliations:** 1 Ph.D. Program for the Clinical Drug Discovery from Botanical Herbs, College of Pharmacy, Taipei Medical University, Taipei, Taiwan; 2 Graduate Institute of Pharmacognosy, College of Pharmacy, Taipei Medical University, Taipei, Taiwan; 3 National Research Institute of Chinese Medicine, Ministry of Health and Welfare, Taipei, Taiwan; 4 School of Pharmacy, College of Pharmacy, Taipei Medical University, Taipei, Taiwan

## Abstract

Targeting cellular mitosis in tumor cells is an attractive cancer treatment strategy. Here, we report that B220, a synthetic benzenesulfonamide compound, could represent a new mitotic inhibitor for the treatment of colorectal cancer. We examined the action mechanism of B220 in the colorectal carcinoma HCT116 cell line, and found that treatment of cells with B220 caused cells to accumulate in G2/M phase, with a concomitant induction of the mitotic phase markers, MPM2 and cyclin B1. After 48 h of B220 treatment, cells underwent apoptotic cell death via caspase-3 activation and poly(ADP ribose) polymerase (PARP) cleavage. In addition, B220 inhibits autophagy by blocking conversion of microtubule-associated protein 1 light chain 3 (LC3-I) to LC3-II and inhibiting autophagic flux. Notably, blockade of autophagy by pharmacological inhibition or using an Atg5-targeting shRNA reduced B220-induced cytotoxicity. Conversely, the autophagy inducer NVP-BEZ235 shows a synergistic interaction with B220 in HCT116 cells, indicating autophagy was required for the observed cell death. In summary, these results indicate B220 combined with the induction of autophagy using the dual PI3K/mTOR inhibitor, NVP-BEZ235, might be an attractive strategy for cancer therapy, and provides a framework for further development of B220 as a new therapeutic agent for colon cancer treatment.

## Introduction

Anti-mitotic agents have been used clinically to treat cancer for decades^1,2^. These chemotherapeutic drugs are designed to disrupt cancer cell microtubule dynamics and cause cell-cycle arrest, thereby inhibiting the hyperproliferative status of these cells and subsequently inducing cell death^3^. Although unwanted side effects of anti-mitotic drugs have been considered a key problem in the clinic, the impressive success of these agents against a variety of malignancies and the valuable scientific insights gained highlight their continuing importance in human diseases^4–7^. As with many antitumor drugs, the mechanism of action of anti-mitotic drugs involves the induction of cell cycle arrest at G2/M phase accompanied by Cdk1/cyclin B1 complex activation^8^. Induction of aberrant mitosis in tumor cells is frequently followed by significant apoptotic cell death^9^. Apoptosis is classified as Type I programmed cell death (PCD) and is mainly characterized with DNA fragmentation and chromatin condensation^10^. Autophagy, recognized as Type II PCD, is characterized by autophagosome formation and subsequent fusion with lysosomes, and serves to eliminate cellular proteins and cytoplasmic organelles^11^. It has been reported that autophagy is associated with different human pathologies, including cancer and neurodegenerative diseases^12,13^. Several studies have shown that autophagy is critical in the regulation of cancer progression and in determining the response of malignant cells to anticancer therapy^14,15^. The central regulator of autophagy is the mammalian target of rapamycin (mTOR) pathway, which, when activated, negatively regulates autophagy to inhibit formation of autophagosomes^16^. Conversely, autophagy-related gene (Atg)-6, also known as beclin-1, can initiate autophagy by associating with vacuolar sorting protein 34 (Vps34), a class III phosphoinositide 3-kinase (PI3K), to recruit other Atg products that are essential for autophagosome formation^17^. During autophagy initiation, the Atg5-Atg12-Atg16 complex promotes the conversion of cytosolic protein light chain 3 (LC3-I) to the membrane-bound form, LC3-II, through lipidation^18^. Thus, autophagy could potentially be suppressed by Atg5 inactivation or pharmacological inhibition with the class III PI3K inhibitor wortmannin^19^. In contrast, inhibition of mTOR by rapamycin blocks the interaction of Atg13 with ULK1 (unc-51 like autophagy-activating kinase 1) to activate the autophagy pathway^19,20^. Many anticancer agents, including temozolomide, camptothecin, ionizing radiation and anti-mitotic drugs, have been reported to induce the autophagy pathway in cells^21–24^. Importantly, it has been demonstrated that modulation of the autophagy pathway can potentiate the cytotoxicity of anticancer therapeutics against malignant cells^22,25,26^.

Here, we identified B220 [7-(4′-cyanophenyl) indoline-1-benzenesulfonamide] as a potent mitotic inhibitor that causes cell cycle arrest and significant cytotoxicity in HCT116 colorectal cancer cells. Our findings indicate that B220 inhibits autophagic activity and acts synergistically in combination with an autophagy inducer to enhance apoptotic cell death.

## Results

### B220 suppresses cell growth and colony formation in HCT116 colorectal cancer cells

**Figure 1:**
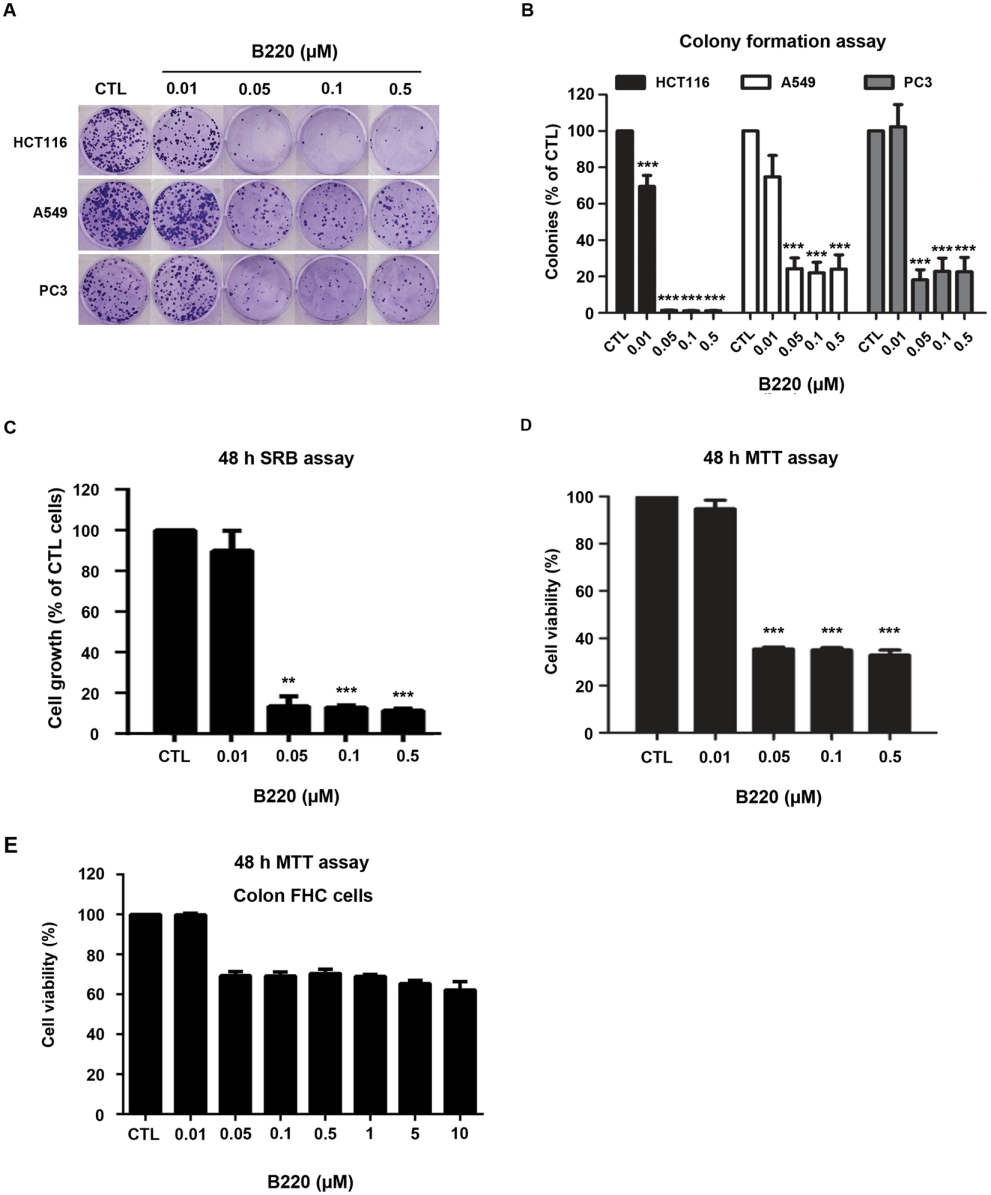
B220 suppresses colony formation and cell viability in colorectal cancer HCT116 cells. (**A**,**B**) Representative images showing B220-mediated inhibition of colony formation inhibits by three different tumor cells lines (**A**) and quantification of colony numbers (n = 3), expressed as a percentage of controls (**B**). (**C**) B220 significantly inhibited the growth of HCT116 cells. Cells were incubated with or without the indicated concentrations of B220 for 48 h, and cell growth was evaluated by SRB assay. (**D**) Effects of B220 on the viability of A549 cells. (**E**) Viability of normal colon FHC cells following treatment with B220. Cells were treated with DMSO (CTL) or different concentrations of B220 for 48 h, and cell viability was analyzed by MTT assay. Data are expressed as means ± S.D. of at least three independent experiments. *P < 0.05; **P < 0.01; and ***P < 0.001 compared with the control group.

To determine the *in vitro* antitumor activity of B220, we performed colony-formation assays using several cancer cell lines. As shown in Fig. 1A and B, B220 exerted an inhibitory effect on the colony-forming abilities of drug-treated cells, suggesting irreversible growth arrest and reproductive cell death. Notably, this cell-killing effect of B220 was more prominent in HCT116 colorectal cancer cells than in prostate cancer PC3 and non-small-cell lung cancer A549 cells (Fig. 1A and B), making HCT116 cells an appropriate model for assessing the effects of B220 and its underlying molecular mechanism of action. Subsequent SRB (sulforhodamine B) and MTT [3-(4,5-dimethylthiazol-2-yl)-2,5-diphenyltetrazolium bromide)] assays in HCT116 cells treated with different concentrations of the drug revealed that B220 suppressed the proliferation of HCT116 cells with submicromolar IC_50_ values (Fig. 1C and D). However, treatment with B220 at high concentration (10 μM) did not cause severe cytotoxicity toward normal colon FHC cells (Fig. 1E). Together, our findings show that B220 exerts significant cytotoxicity against HCT116 colorectal cancer cells and show selectivity for cancer cells.

### B220 induces mitotic arrest followed by apoptosis in HCT116 colorectal cancer cells

**Figure 2:**
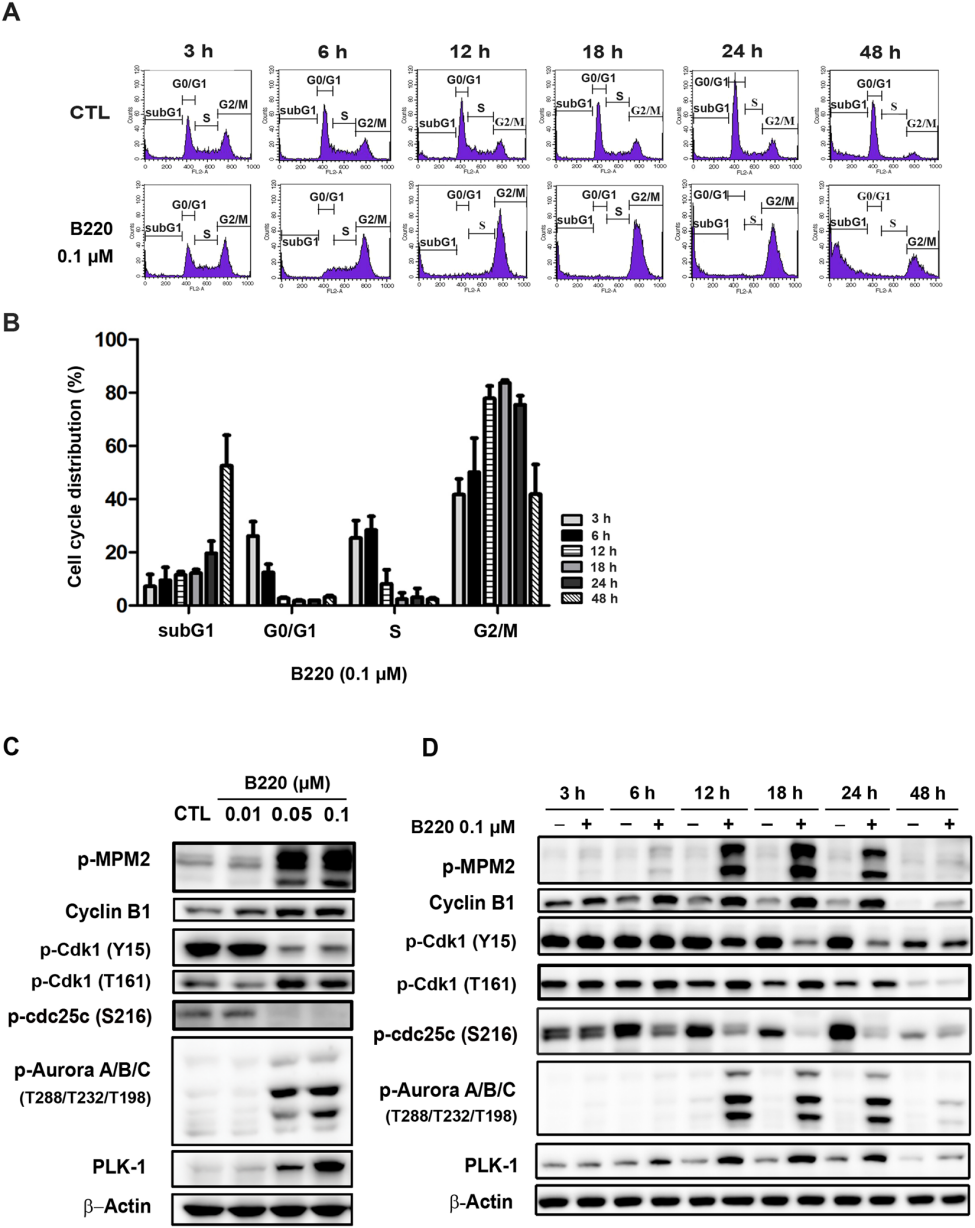
B220 induces mitotic arrest in HCT116 cells. (**A**,**B**) Time-dependent effects of B220 on G2/M phase arrest in HCT116 cells. Cells were treated with DMSO or B220 (0.1 μM) for the indicated times, and the cell cycle distribution was analyzed by flow cytometry. Quantitative data (**B**) are based on flow cytometry histograms, and are presented as means ± S.E.M. of at least three independent experiments that yielded similar results. (**C**,**D**) Concentration-dependent (**C**) and time-dependent (**D**) effects of B220 on the induction of mitotic arrest. Cells were treated with the indicated concentrations of B220 for the indicated times, and cell lysates were immunoblotted using the indicated antibodies.

To investigate the mechanism underlying B220-induced repression of cell growth, we examined the effects of B220 on cell cycle progression using flow cytometry. Cells began to accumulate in G2/M phase at 6 h after treatment; after 24 h, this peak shifted from G2/M to an apoptotic subG1 phase (Fig. 2A and B). Consistent with this, expression levels of the general mitotic markers, MPM-2 and cyclin B1, were induced in a concentration-dependent manner (Fig. 2C), peaking between 6 and 24 h after B220 treatment (Fig. 2D). Progression from G2 to M phase is initiated by activation of the Cdk1/cyclin B1 heterodimer complex, whose activity is regulated primarily by the kinase Wee1 and phosphatase Cdc25c, and is maintained from prophase to metaphase^27^. Thus, in addition to significantly increasing MPM2 and cyclin B1 expression, B220 treatment similarly induced activating phosphorylation of Cdk1 residue Thr161, dephosphorylation of the inhibitory Cdk1 residue Tyr15, dephosphorylation of Cdc25c residue Ser216, and phosphorylation of the mitotic kinases PLK1 (Polo-like kinase-1) and Aurora kinase A/B/C, all of which also play distinct roles in regulating cell division^27–29^. As shown in Fig. 2C and D, B220 increased the levels of phospho-Cdk1(T161), PLK1 and phospho-Aurora kinase, while decreasing the levels of phospho-Cdk1 (Y15) and phospho-Cdc25c (S216). Furthermore, the similar effects can be detected in different cancer cell lines (Supplementary Fig. 1), indicating B220 activates the Cdk1/cyclin B1 complex in cancer cells.

**Figure 3:**
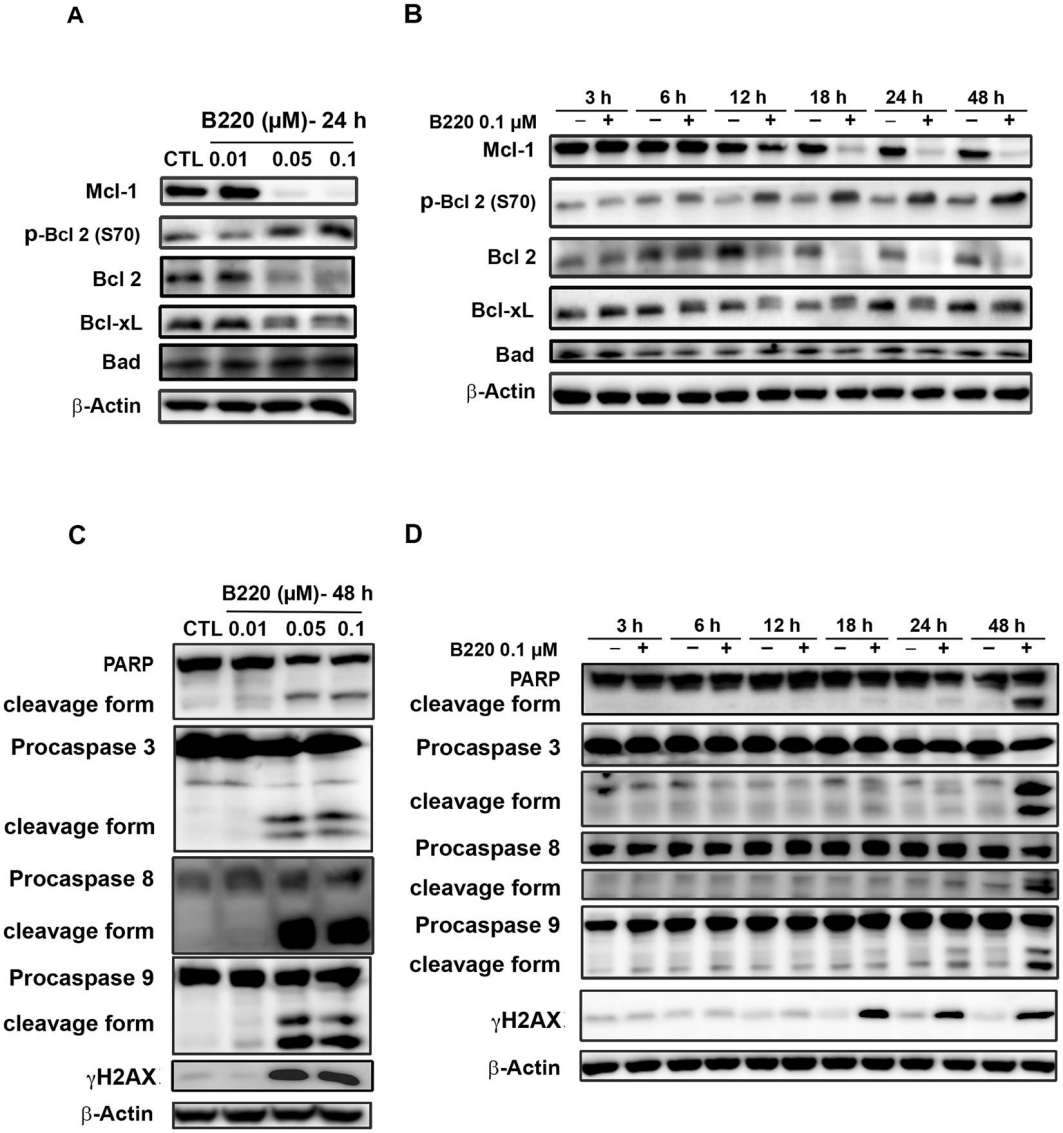
B220 inhibits anti-apoptotic Bcl-2 family proteins and induces apoptosis in HCT116 cells. (**A**,**B**) Expression levels of anti- and pro-apoptotic Bcl-2 family proteins. (**C**,**D**) Effects of B220 on apoptosis in HCT116 cells. B220 increased levels of the cleaved (activated) forms of PARP, γH2AX, caspase-3, -8, and -9 in a concentration-dependent (**C**) and time-dependent (**D**) manner. HCT116 cells were exposed to B220 (0.1 μM) for the indicated times, and cell lysates were immunoblotted using the indicated antibodies.

The Bcl-2 protein family includes anti- and pro-apoptotic members that control the balance of life-or-death cellular “decisions” by participating in the regulation of mitotic arrest in cells treated with an apoptotic stimulus^30^. Here, we found that expression of the anti-apoptotic proteins, Bcl-2, Mcl-1 and Bcl-_XL_, decreased after 12 h of treatment with B220 (Fig. 3A and B). In contrast, levels of phospho-Bcl-2 (Ser70), which is known to induce apoptosis^31^, were increased by drug treatment (Fig. 3A and B). Expression of Bad, another pro-apoptotic protein, was not appreciably altered by B220 in HCT116 cells (Fig. 3A and B). Notably, the subG1 phase showed a dramatic induction after 48 h of treatment (Fig. 2B) in parallel with significant activation of caspase-3, -8, and -9, and poly-(ADP-ribose) polymerase (PARP) (Fig. 3C and D). Taken together, these results indicate that B220 induces cell cycle arrest at the G2/M phase, inhibits anti-apoptotic proteins, and triggers apoptotic cell death.

### B220 inhibits autophagosome formation and decreases autophagic flux in cells

**Figure 4:**
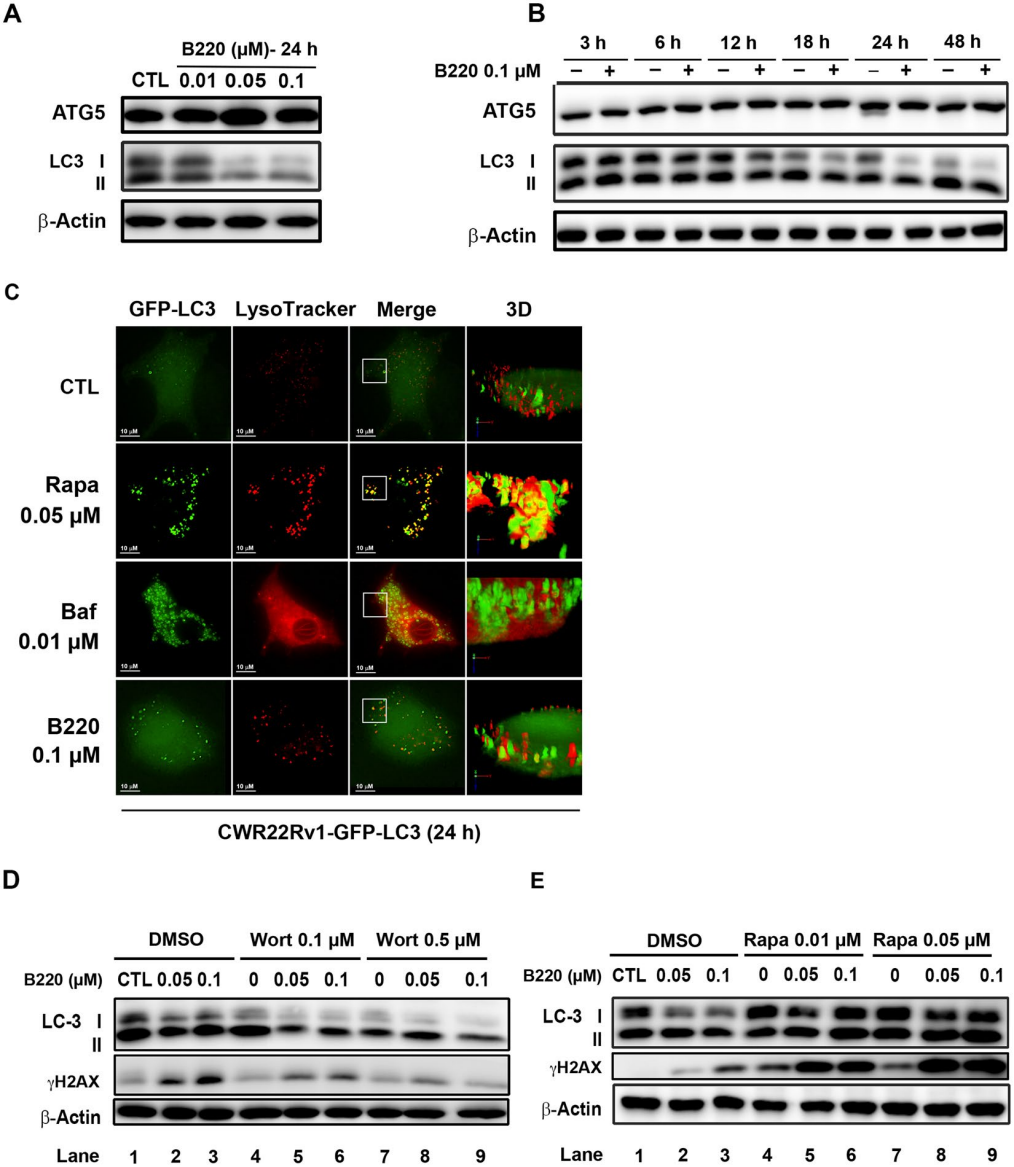
B220 inhibits autophagy in HCT116 cells. (**A**,**B**) Concentration-dependent (**A**) and time-dependent (**B**) effects of B220 on the conversion of endogenous LC3-I to LC3-II. Cells were treated with different concentrations of B220 for the indicated times, and cell lysates were analyzed by Western blotting. (**C**) Microscopic analyses of the effects of B220 on the pattern of GFP-LC3 and lysosome fluorescence. CWR22Rv1-GFP-LC3 cells (green) were treated with B220 for 24 h. Lysosomes were labeled with LysoTracker (red) at 37 °C for 30 min before live-cell imaging using a confocal microscope. Scale bars: 10 μm. (**D**,**E**) Effects of combined treatment with B220 and the autophagy inhibitor wortmannin (**D**) or autophagy activator rapamycin (**E**) in HCT116 cells. Cells were treated with B220 for 24 h in the presence or absence of wortmannin or rapamycin, and cell lysates were analyzed by Western blotting.

It is well known that autophagy plays important roles in cellular functions by degrading cytoplasmic components via the lysosome-dependent pathway^32^. Several reports have shown that mitotic inhibitors can activate pro-cell death or pro-survival autophagy depending on cellular context^33,34^. Here, we examined the ability of B220 to facilitate the conversion of LC3-I to LC3-II, an essential step in autophagosome formation, and promote the expression of Atg5, which is responsible for autophagy initiation^18^, in colorectal HCT116 cells. As shown in Fig. 4A, B220 decreased LC3-I protein expression levels without changing Atg5 levels, suggesting that B220 exerts an inhibitory effect on autophagy. To further elucidate the effects of drug treatment on autophagosome formation and the association between autophagosomes and lysosomes, we analyzed the colocalization of autophagosomes and lysosomes in CWR22Rv1 cells stably expressing a fusion protein of green fluorescent protein (GFP) and LC3 (GFP-LC3) using deconvolution fluorescence microscopy. Rapamycin, which induces autophagosome formation, and bafilomycin A1, which blocks fusion of autophagosomes with lysosomes, were included as reference compounds. As shown in Fig. 4B, rapamycin induced formation of GFP-LC3 puncta, as evidenced by the clear presence of green dots in cells. Importantly, some colocalization of autophagosomes and lysosomes (yellow dots) was also observed, indicating that induction and degradation of autophagosomes occurred simultaneously in rapamycin-treated cells. In contrast, bafilomycin A1-treated cells exhibited accumulation of autophagosome puncta and significant induction of lysosome (LysoTracker staining), but colocalization of autophagosome and lysosome was barely detectable, reflecting block of autophagosome fusion with lysosomes. Interestingly, cells treated with B220 did not show an appreciable change in autophagosome (GFP-LC3 puncta) accumulation in cells compared with control cells, suggesting that B220 did not induce autophagy. Using a three-dimensional (3D) analysis to further clarify the interaction between autophagosomes and lysosomes, we found that B220 treatment interfered the fusion of autophagosomes and lysosomes, as evidenced by the lack of association between green and red dots (Fig. 4C). Taken together, these findings suggest that B220 is an autophagy inhibitor. To further investigate the role of autophagy in B220-induced cytotoxicity, we used a pharmacological approach, blocking autophagy with the class III PI3K inhibitor wortmannin and inducing autophagy with the mTOR inhibitor rapamycin^19^. Wortmannin-induced inhibition of class III PI3K resulted in a decrease in LC3-I (Fig. 4D, lane 7), whereas rapamycin-induced autophagy mediated by inhibition of mTOR caused LC3-I and -II formation (Fig. 4E, lane 7). Cotreatment with B220 further potentiated the wortmannin-induced reduction in LC3- I expression (Fig. 4D, lane 8 and 9) and rapamycin-induced increase in LC3-I formation (Fig. 4E, lane 8 and 9), suggesting that B220 inhibits autophagosome formation under both autophagy-activated and -inactivated scenarios. Notably, the combination of B220 with the autophagy inhibitor wortmannin for 24 h attenuated the B220-induced accumulation of γH2AX, a cellular marker of DNA-double strand breaks (Fig. 4D). In contrast, the combination of B220 with the autophagy inducer rapamycin potentiated B220-induced formation of γH2AX (Fig. 4E). These findings suggest that targeting the modulation of autophagy plays an important role in B220-induced cell death in HCT116 cells, in which simultaneous induction and inhibition of autophagy may enhance B220-induced apoptosis.

### Atg5 knockdown reduces B220-induced apoptosis in HCT116 cells

**Figure 5:**
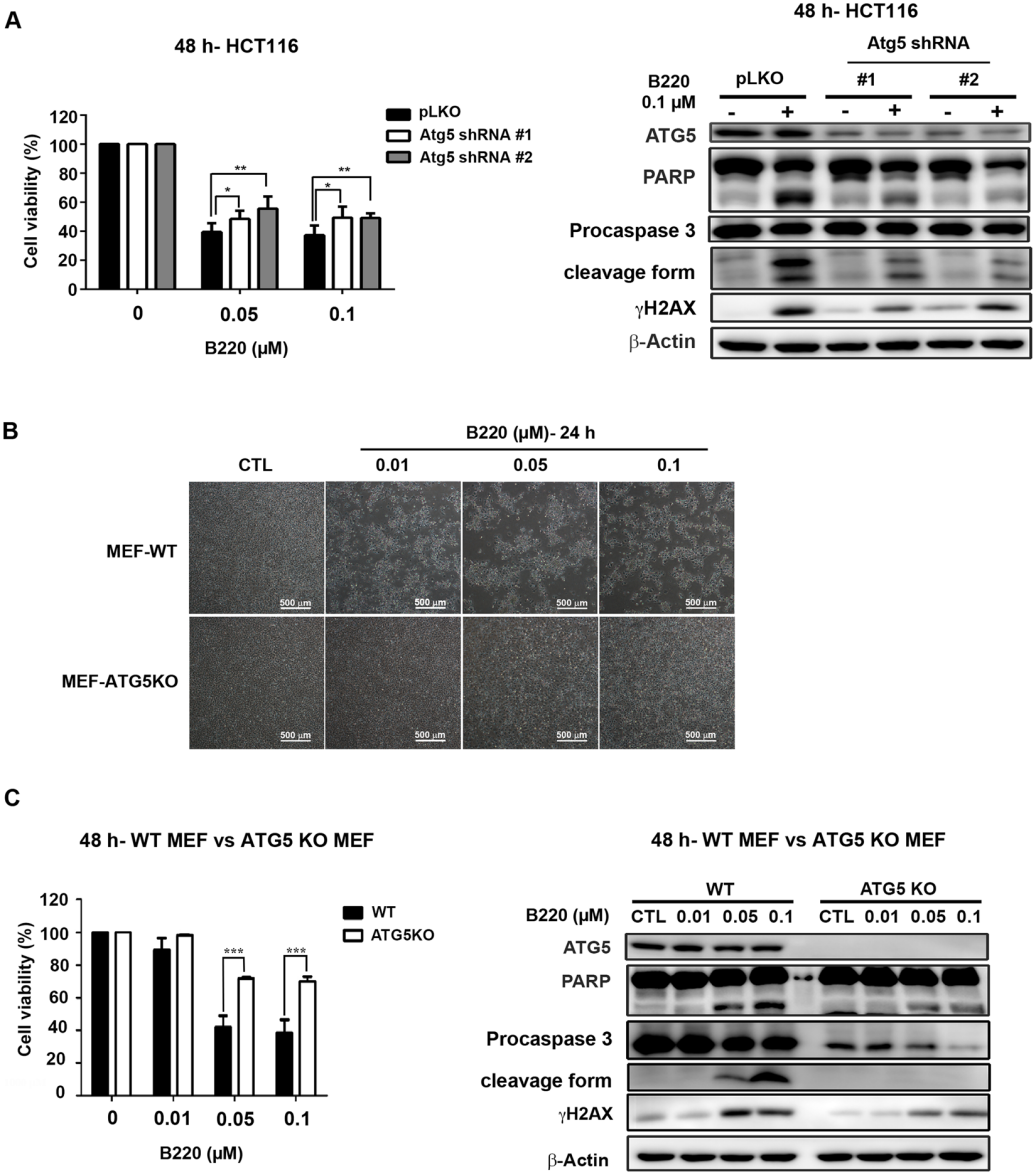
Blockade of autophagy reduces B220-induced cytotoxicity. (**A**) HCT116 cells stably expressing a pLKO vector or pLKO-shAtg5 were treated with the indicated concentrations of B220 for 48 h. Cytotoxicity was analyzed by MTT assay (left), and protein lysates were analyzed Western blotting using the indicated antibodies (right). (**B**) Effects of B220 on cell numbers in wild-type MEFs and Atg5-KO MEFs. Cells were treated with different concentrations of B220 for 24 h, and images from different treatment groups were captured. Scale bars: 100 *μ*m. (**C**) Effects of B220 on cell viability (left) and apoptotic marker expression (right) in wild-type MEFs and Atg5-KO MEFs. Cells were treated with different concentrations of B220 for 48 h. Cytotoxicity was evaluated by MTT assay (left), and Western blot analyses were conducted using the indicated antibodies (right).

To further investigate the role of autophagy in B220-induced cell death, we inhibited autophagy initiation in HCT116 cells by knocking down Atg5. As shown in Fig. 5A, knockdown (KD) of Atg5 expression rescued B220-induced suppression of cell viability (left panel) and reversed B220-induced accumulation of γH2AX and activation of PARP and caspase 3 (right panel), suggesting that the Atg5 autophagy pathway enhances B220-induced cell death. Moreover, treatment with B220 for 24 h lead to a dramatic decrease in cell numbers in wild-type mouse embryo fibroblasts (MEFs), without causing an obvious change in cellular morphology or cell death in Atg5-knockout (KO) MEFs (Fig. 5B). Cell viability was similarly significantly increased in B220-treated Atg5-KO MEFs compared with wild-type MEFs following treatment for 48 h (Fig. 5C, left panel). Likewise, B220-induced apoptotic cell death was significantly reduced in Atg5-KO MEFs (Fig. 5C, right panel). Taken together, these findings indicate that inhibition of autophagy through deprivation of Atg5 greatly reduces B220-induced cell death.

### Autophagy induction with the combined PI3K and mTOR inhibitor NVP-BEZ235 potentiates B220-induced cell death

**Figure 6:**
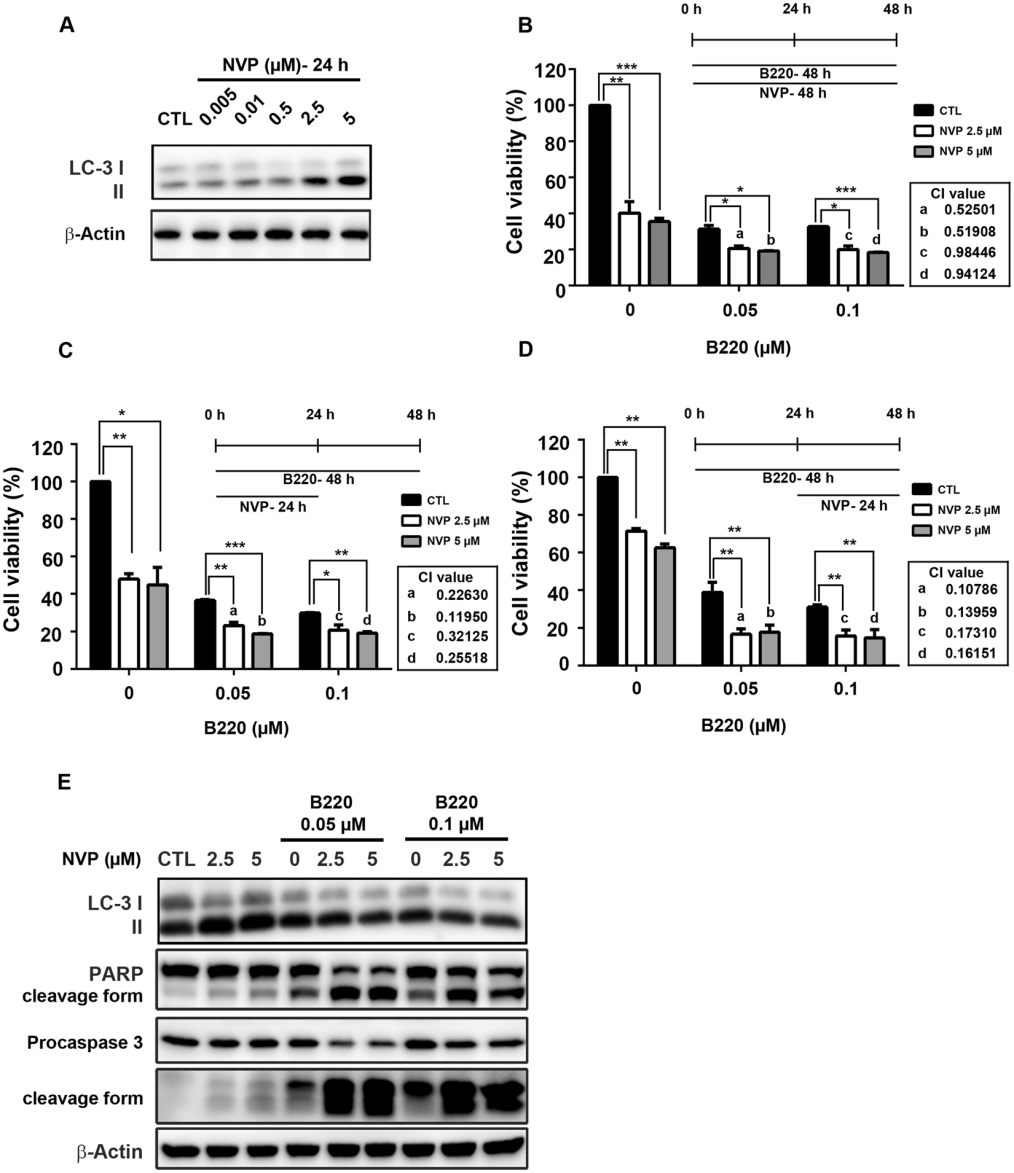
The autophagy inducer NVP-BEZ235 enhances B220-induced cytotoxicity in HCT116 cells. (**A**) NVP-BEZ235 (NVP) induced the conversion of endogenous LC3-I to LC3-II. (**B**–**D**) Synergistic effects of three different schedules of B220 and NVP-BEZ235 treatment on cytotoxicity in HCT116 cells. Cells were treated with the indicated compounds at the indicated concentrations for 48 h, and cytotoxicity was evaluated by MTT assay. (**E**) Apoptotic effects of B220 plus NVP-BEZ235. Cells were treated using the combination exposure schedule shown in (**D**), and protein lysates were analyzed by Western blotting using the indicated antibodies.

Our findings suggest the possibility that combining B220 with an autophagy inducer would enhance B220-induced cell death effects. To test this, we cotreated cells with B220 and the dual PI3K/mTOR inhibitor, NVP-BEZ235. Both 2.5 and 5 µM NVP-BEZ235 induced significant autophagy in HCT116 cells after a 24-h treatment, as evidenced by LC3-II formation (Fig. 6A). An assessment of the effect of different dosing schedules of B220 and NVP-BEZ235 on the viability of HCT116 cells showed that, compared with B220 or NVP-BEZ235 alone, treatment with B220 plus NVP-BEZ235 with all three exposure-schedules decreased viability (Fig. 6B–D). In each case, combination index (CI) values were less than 1, indicating a synergistic interaction between B220 and NVP-BEZ235. Cotreatment with B220 and NVP-BEZ235 for 48 h resulted in CI values ranging from 0.5 to 0.9 (Fig. 6B, a–d). These CI values were improved to 0.1–0.3 by cotreatment with B220 and NVP-BEZ235 for 24 h followed by B220 alone for a total of 48 h (Fig. 6C, a–d). Notably, B220 for 24 h followed by addition of NVP-BEZ235 for a total of 48 h exerted the greatest synergy; under these conditions, all CI values were approximately 0.1 (Fig. 6D, a–d), indicating that exposure to B220 before NVP-BEZ235 produces the most effective synergistic interaction in HCT116 cells. Subsequent Western blot analyses revealed that this optimal combination therapy dosing with B220 and NVP-BEZ235 significantly potentiated apoptosis. As shown in Fig. 6E, NVP-BEZ235 alone induced autophagy without causing obvious activation of PARP or caspase-3, whereas combination treatment increased responsiveness to B220 in association with significant activation of PARP and caspase-3. Collectively, these findings show that B220 inhibits autophagy pathways in colorectal cells, and that combination therapy with the autophagy inducer NVP-BEZ235 enhances B220 cytotoxicity.

## Discussion

Previous studies have shown that various anti-mitotic agents possess promising, wide-spectrum activity against different types of malignancies^1,35^. Given the many challenges posed by the development of drug resistance during therapy, the US Food and Drug Administration (USFDA) has approved some novel anti-mitotic therapeutic agents for restoring efficacy lost due to drug resistance (e.g., ixabepilone and eribulin), and a number of ongoing studies seek to achieve better clinical values so as to solve existing problems^36–38^. B220 has been shown to display effective antiproliferative activity against numerous cancer cell lines, including multidrug resistant (MDR)-positive cells, which overexpress P-glycoprotein 170 (p-gp170)/MDR^39^. In addition, B220 causes tubulin depolymerization and shows strong binding affinity for the colchicine-binding domain^39^. In this study, we clearly showed that B220 causes cell cycle arrest at G2/M phase and apoptotic cell death in HCT116 colorectal cancer cells. Parallel results obtained from flow cytometry analyses showed that B220 activates mitotic markers, including cyclin B1 and MPM2. Moreover, increased levels of Thr161-phosphorylated (activated) Cdk1 and decreased levels of Tyr15-phosphorylated (inhibited) Cdk1 indicate that accumulation of activated Cdk1 in cells peaks after 18 h of treatment with B220. Our data further showed a close correlation between an altered cell cycle distribution, analyzed by flow cytometry (Fig. 2B), and mitotic arrest, determined by Western blotting (Fig. 2D).

Our results further showed downregulation of pro-survival Bcl-2 and induction of Bcl-2 phosphorylation by B220. In addition to the well-known role of Bcl-2 family proteins in regulating permeabilization of the mitochondrial outer membrane^40^, the role of phosphorylation of Bcl-2 at Ser70 has been widely discussed. It has been demonstrated that CDK1 may be responsible for phosphorylation of Bcl-2 at Ser70, which augments the antiapoptotic effect of Bcl-2^31^. In addition, it has been demonstrated that the Bcl-2 Ser70 phosphorylation site is required to maintain the tight association between Bax and Bcl-2 necessary to prevent apoptosis^41^. However, it has also been shown that blocking Bcl-2 Ser70 phosphorylation by expressing a Bcl-2 Ser70 mutant failed to protect against histone deacetylase (HDAC) inhibition-induced cell death^42^. Further, several lines of evidence support the conclusion that Bcl-2 phosphorylation is simply a biomarker of anti-mitotic agents^43,44^, suggesting that the consequences of Bcl-2 phosphorylation might differ depending on cell type and stimulus.

It is well-known that microtubule-targeting agents induce apoptosis through JNK activation-dependent pathway. In our study, treatment with B220 for 24 h induces expression level of phospho-JNK (T183/Y185) in cells (Supplementary Fig. 2A). Combination with JNK inhibitor SP600125 reverses B220-induced expression of markers for G2/M cell cycle arrest (Supplementary Fig. 2A). In addition, cotreatment with JNK inhibitor for 48 h also attenuates B220-induced apoptotic cell death (Supplementary Fig. 2B). These results suggest activation of JNK contributes to B220 induced- G2/M cell cycle arrest and apoptosis in HCT116 cells. However, B220 combined with JNK inhibitor did not change LC3 I expression level (Supplementary Fig. 2A), suggesting JNK activation did not play a major role in autophagy pathway.

Autophagy is an important catabolic pathway that is essential for maintaining cellular homeostasis^45^. The most common indicator of whether cells are undergoing activated autophagy signaling is the formation of double-membrane autophagic vesicles (autophagosomes), which can be detected by fluorescence microscopy in GFP-LC3–expressing cells^46^. However, an increase in the number of autophagosomes (i.e., green GFP puncta in GFP-LC3–expressing cells) may represent either autophagy activation or inhibition of a downstream step in autophagy that blocks fusion of autophagosomes and lysosomes^17^. Therefore, quantifying GFP-LC3 puncta is insufficient for evaluating autophagy activation. To carefully investigate B220-induced autophagic activity, we determined the “autophagic flux” by comparing the effects of the autophagy activator rapamycin and autophagy inhibitor bafilomycin A1, the latter of which blocks the fusion of autophagosomes and lysosomes, using deconvolution fluorescence microscopy (Fig. 4B). We clearly observed significant GFP puncta, indicating autophagosome formation, likely reflecting autophagy induction, in rapamycin- and bafilomycin A1-treated cells. However, LysoTracker staining in combination with an analysis of GFP puncta revealed that rapamycin increased autophagic flux by promoting fusion of autophagosomes and lysosomes (Fig. 4B, yellow), whereas bafilomycin A1 reduced autophagic flux, as evidenced by the presence of distinct autophagosomes and lysosomes in 3D images (Fig. 4C). Our findings showed that B220 decreases autophagic flux (Fig. 4C) and expression level of LC3-I protein (Fig. 4A and B), indicating that treatment with B220 inhibits autophagic activity in cells. In addition to formation of LC3-II and autophagosomes, degraded P62 is another biomarker for the detection of autophagy induction^47^. P62 is recruited with LC3-II to autophagosomes and is further degraded within the autolysosome to complete the autophagy pathway^48^. Further, it has been reported that microtubule destabilizers inhibit the fusion of autophagosomes and lysosomes, a microtubule-dependent step required to complete the autophagy process^49,50^. Given this, the expectation is that B220 would increase p62 accumulation in cells. Interestingly, however, B220 did not significantly alter p62 in HCT116 cells, but did induce a band shift after a 24-h treatment (Supplementary Fig. 3), suggesting that B220 causes a posttranslational modification of P62. Previous reports have shown that p62 is not only targeted for degradation by autophagy, but also participates in critical cellular functions through the regulation of physiologically important signaling factors that control diseases, such as cancer and insulin resistance^51,52^. It has been shown that phosphorylation of p62 at Thr269 and Ser272 by CDK1 maintains the activity of CDK1 necessary to regulate exit from mitosis^53^. Our findings revealed that B220-induced p62 phosphorylation can be blocked by cotreatment with the CDK1 inhibitor roscovitine, indicating B220 triggers CDK1-mediated p62 phosphorylation at Thr 269 and Ser272 (Supplementary Fig. 3).

The role of autophagy in cancer has been widely discussed, and considerable evidence has shown that inhibition of autophagy may enhance the effects of anticancer agents^34,54,55^. We found that combining B220 with the autophagy inhibitor wortmannin decreased γH2AX formation (Fig. 4D). In contrast, the autophagy inducer rapamycin enhanced B220-induced γH2AX formation (Fig. 4E), suggesting that autophagy activation potentiates the anticancer activity of B220. To circumvent non-specific effects of pharmacological inhibitors, we next used Atg5-KD and Atg5-KO cells to investigate the role of autophagy in the anticancer activity of B220. These experiments showed that the ability of B220 to induce apoptotic cell death was greatly diminished in Atg5-KD HCT116 cells and Atg5-KO MEFs (Fig. 5A–C). Moreover, knockdown of Atg5 greatly reduced B220-induced expression of markers for G2/M cell cycle arrest (Supplementary Fig. 4) and cytotoxicity (Fig. 5A). It is noteworthy that Atg5-KO MEFs provided better protection against B220-reduced cell viability than Atg5-KD HCT116 cells did, suggesting that a basal level of Atg5-mediated autophagy may participate in B220-induced cell death.

NVP-BEZ235 has been reported as a dual PI3K/mTOR inhibitor that induces autophagy and exerts anticancer activity against several cancer cell types^56,57^. Previous studies have reported that different exposure schedules of drug combinations cause different results^58,59^, suggesting that schedule-dependent interactions between anticancer treatments should be studied carefully. Our current findings indicate that the combination of B220 and NVP-BEZ235 resulted in synergistic effects using three different exposure schedules (Fig. 6B–D). Notably, exposure to B220 before NVP-BEZ235 led to the most promising synergistic interaction in HCT116 cells (Fig. 6D and E), suggesting that the mechanism by which NVP-BEZ235 potentiates B220 cytotoxicity is related to the timing of autophagy initiation. It has been reported that appropriate modulation of autophagy by inhibiting cytoprotective autophagy or enhancing pro-death autophagy could potentiate the cytotoxicity of anticancer therapy in malignant cells^22,25,26^. Although previous studies have shown that the cytotoxicity of NVP-BEZ235 is enhanced by cotreatment with an autophagy inhibitor, owing to NVP-BEZ235-induced cytoprotective autophagy^56,57,60^, NVP-BEZ235 has been demonstrated to induce pro-death autophagy in urothelial carcinoma cells^61^, suggesting that the outcome—pro-survival or pro-death autophagy—may be context dependent.

In summary, the current findings indicate that B220 is a highly effective anti-mitotic agent that reduces cell viability and autophagic activity in HCT116 cells. Autophagy modulation by combined treatment with B220 and the autophagy inducer NVP-BEZ235 resulted in synergy between the two agents that significantly enhanced cytotoxicity and dramatically induced apoptotic cell death. Induction of autophagy in the context of a B220-induced reduction in autophagic activity may produce greater cellular stress, which in turn potentiates cell death. These results indicate that activation of autophagy might increase the efficacy of B220, suggesting a potential therapeutic strategy for enhancing the chemotherapeutic efficacy of this drug.

## Methods

### Cell lines and reagents

A549, PC3, HCT116, and normal intestinal epithelial fetal human colon (FHC) cells were obtained from the American Type Culture Collection (ATCC) (Manassas, VA, USA). Cells were maintained in 10% fetal bovine serum (FBS)-supplemented RPMI 1640 medium or DMEM/F12 medium (GIBCO, Grand Island, NY, USA) and 1% penicillin–streptomycin (GIBCO) at 37 °C in a humidified incubator containing 5% CO2. B220, 7-(4′-Cyanophenyl) indoline-1-benzenesulfonamide, was obtained from Professor Jing-Ping Liou (College of Pharmacy, Taipei Medical University, Taiwan). Antibodies against various proteins were obtained from the following sources: PARP (Poly-ADP-ribose polymerase), cyclin B1, Bcl-2, Bcl-xL, Mcl-1, PLK-1, Bad, and p62, were obtained from Santa Cruz Biotechnology Inc. (Dallas, TX, USA). Bcl-2 (Ser70), caspase 8, capspase 9, γH2AX, phospho-cdc2 (Tyr15), phospho-cdc2 (Thr161), phospho-cdc25c (Ser216), Phospho-Aurora A (Thr288)/Aurora B (Thr232)/Aurora C (Thr198), and Atg5 were obtained from Cell signaling (Danvers, MA, USA). Beta-actin and MPM-2 were from Millipore (Billerica, MA, USA). Caspase 3 and was obtained from IMGENEX (San Diego, CA, USA). LC3 was purchased from Novus (Littleton, CO, USA). NVP-BEZ235 was obtained from Selleckchem (Houston, TX, USA). Rapamycin and wortmannin were from Cayman Chemical (Ann Arbor, MI, USA). Anti- mouse and anti-rabbit IgGs were from Jackson ImmunoResearch Laboratories (West Grove, PA, USA).

### Cell viability assay

Cells were seeded in 96-well plastic plates and exposed to indicated drugs for 48 h. Cell viability was assayed by the 3-(4,5-dimethylthiazol-2-yl)-2,5-diphenyltetrazolium bromide assay. Growth inhibition was expressed as the percentage of surviving cells in drug-treated versus DMSO-treated control cells (which was considered as 100% viability). The IC_50_ value was the concentration resulting in 50% cell growth suppression by a 48-h exposure to drug(s) compared with untreated control cells. Interactions between B220 and NVP-BEZ235 were expressed as the combination index by the CompuSyn software: <1 represents synergistic cytotoxicity; = 1 represents addictive cytotoxicity; and >1 represents antagonistic cytotoxicity^62^.

### SRB (sulforhodamine B) assay

Cells were seeded into 96-well plates and cultured overnight followed by the exposure to gradient concentrations of B220 for 48 h. Cells were then fixed *in situ* with 10% trichloroacetic acid (TCA) to represent a measurement of the cell population at the time of drug addition (T0). After an additional 48 h incubation with or without B220 in medium with 5% FBS, the assay was terminated by 10% TCA. SRB dye pursed from Sigma (St. Louis, MO, USA) at 0.4% (w/v) in 1% acetic acid was added to stain the cells. Unbound dye was removed by 1% acetic acid and the plates were air dried. Bound dye was subsequently solubilized with 10 mM trizma base, and the absorbance was read at a wavelength of 515 nm^63^.

### Clonogenic assay

Cells were plated in 96 well (5000 cells per well) and exposed to DMSO or B220 at indicated concentrations for 24 h. The drugs were then washed away and cells were trypsinized and seeded in 6-well plates (500 cells/well) for continuing growing for 14 days. The colonies were fixed and stained with crystal violet (0.5% in 70% ethanol) and the experiments were repeated at least twice.

### FACScan Flow Cytometric analysis

Cells were seeded in 6-well plates (2.5 × 10^5^/well) and treated with DMSO or B220 at various concentrations for indicated times. Cells were washed with phosphate-buffered saline, fixed in ice-cold 70% ethanol at −20 °C overnight, and stained with propidium iodide (80 ug/ml) containing Triton X-100 (0.1%, v/v) and RNase A (100 ug/ml) in phosphate-buffered saline. DNA content was analyzed with the FACScan and CellQuest software (Becton Dickinson, Mountain View, CA, USA).

### Immunoblotting and lentivirus expression system

Cells were seeded in dishes and allowed to attach for overnight. The cells were treated with drugs at indicated concentrations for indicated times. After the indicated exposure time, cells were lysed and the immunoblotting was performed as previous described^63^. Lentiviral shRNA expression plasmids of shAtg5 (TRCN0000151474, TRCN0000151963) were purchased from the National RNAi Core Facility (Academia Sinica, Taipei, Taiwan).

### Microscopic image acquisition

For deconvolution microscopy, GFP-LC3-expressing CWR22-Rv1 cells (a generous gift from Mr. Chun A. Changou, Taipei Medical University) were seeded and treated with B220 (0.1 μM), Bafilomycin A1 (0.01 μM) or Rapamycin (0.01 μM) for 24 h in a 35 mm glass- bottomed dish (0.17 mm thick). LysoTracker (Thermo Fisher Scientific, Waltham, MA, USA) were used to stain Lysosome. For image captured, live cell was assessed by Wide-field Delta Vision deconvolution microscope (Applied Precision Inc., Eagle, ID, USA), equipped with inverted microscope (IX-71; Olympus, Tokyo, Japan), 100x/1.42 NA oil immersion objective lens, and camera (CoolSnap ES2; Photometrics, Tucson, AZ, USA). Images were reconstructed by using SoftWorx v6.1.1 software (Applied Precision Inc.), and analyzed with Volocity software (PrekinElmer, Waltham, MA, USA), as described previously^64^.

### Statistics and data analysis

Each experiment was performed at least three times, and presentative data are shown. Data in bar graph are given as the means ± S.D. Means were checked for statistical difference using the t-test and *P*-values less than 0.05 were considered significant (*P < 0.05, **P < 0.01, ***P < 0.001).

### Data Availability

The datasets generated during and/or analysed during the current study are available from the corresponding author on reasonable request.

## Electronic supplementary material

Supplementary Info
