# Supplementary material for: Identification of 7-(4′-Cyanophenyl)indoline-1-benzenesulfonamide as a mitotic inhibitor to induce apoptotic cell death and inhibit autophagy in human colorectal cancer cells
Source: Sci Rep. 2017 Sep 29;7:12406. doi: 10.1038/s41598-017-12795-5 (PMC5622076; doi:10.1038/s41598-017-12795-5)

Identification of 7-(4'-Cyanophenyl)indoline-1-benzenesulfonamide as a mitotic inhibitor to induce apoptotic cell death and inhibit autophagy in human colorectal cancer cells

Tung-Yun Wu[a], Ting-Yu Cho[b], Chung-Kung Lu[c],

Jing-Ping Liou[d], Mei-Chuan Chen[a,b,*]

[a] Ph.D. program for the clinical drug discovery from botanical herbs, College of Pharmacy, Taipei Medical University, Taipei, Taiwan

[b]Graduate Institute of Pharmacognosy, College of Pharmacy, Taipei Medical University, Taipei, Taiwan.

[c]Chinese Medicinal Chemistry, National Research Institute of Chinese Medicine, Taipei, Taiwan

[d]School of Pharmacy, College of Pharmacy, Taipei Medical University, Taipei, Taiwan

***Corresponding author:**

Mei-Chuan Chen, Ph.D. program for the clinical drug discovery from botanical herbs, College of Pharmacy, Taipei Medical University, 250 Wuxing Street, Taipei 11031, Taiwan.

E-mail: mcchen1250@tmu.edu.tw

Supplementary Information

Supplementary Fig. 1 Wu et al.

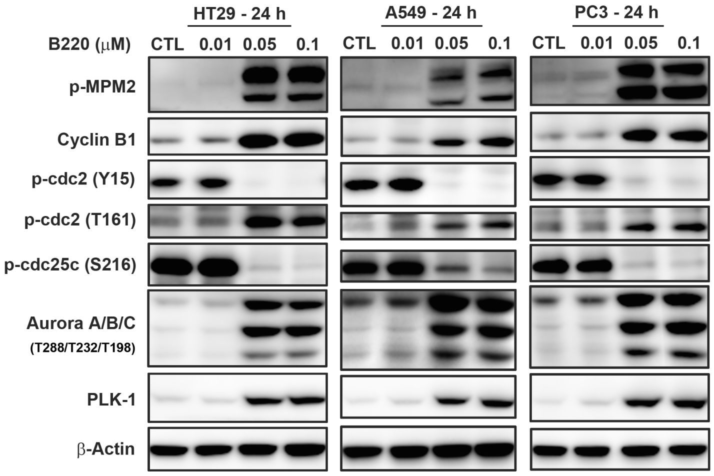

Supplementary Fig. 1. B220 induces mitotic arrest in three different cancer cells. Cells were treated with the indicated concentrations of B220 for 24 h, and cell lysates were immunoblotted using the indicated antibodies.

Supplementary Fig. 2 Wu et al.

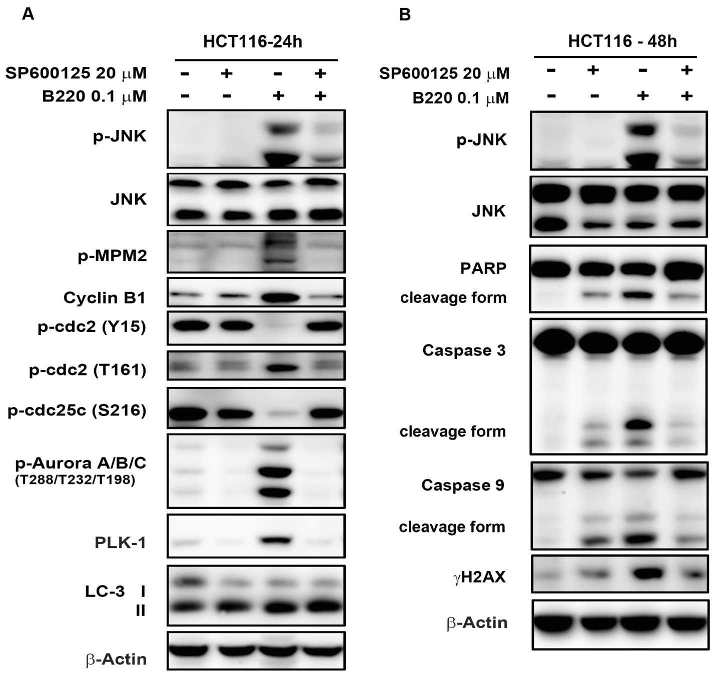

Supplementary Fig. 2. B220 induces JNK-dependent G2/M cell cycle arrest and apoptosis in HCT116 cells. Cells were treated with the indicated concentrations of B220 for indicated times, and cell lysates were immunoblotted using the indicated antibodies.

Supplementary Fig. 3 Wu et al.

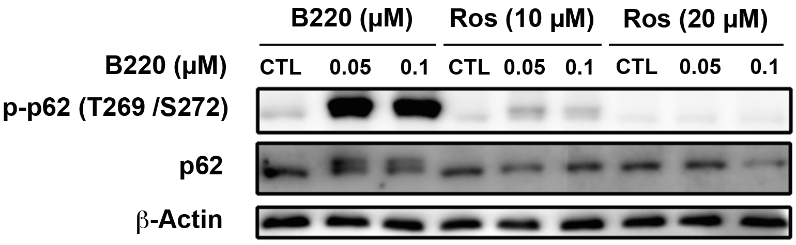

Supplementary Fig. 3. Effects of combined treatment with B220 and roscovitine (ROS) for 24 h in HCT 116 cells. Cells were treated with B220 in the presence or absence of roscovitine (ROS) for 24 h, and cell lysates were analyzed by Western blotting.

Supplementary Fig. 4 Wu et al.

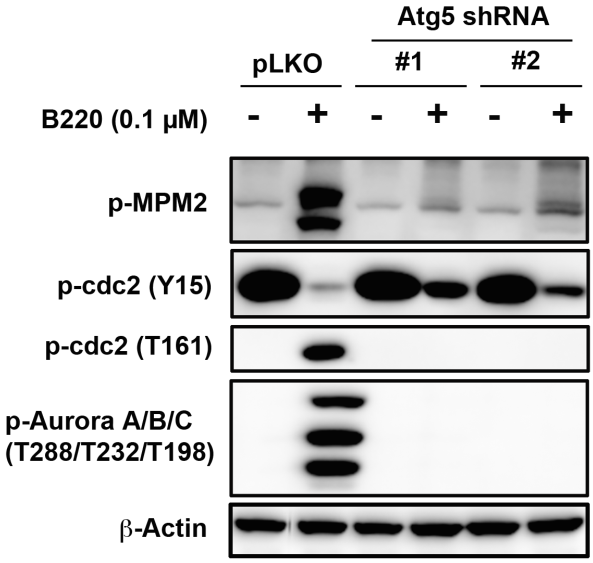

Supplementary Fig. 4. Knockdown of Atg5 reversed B220-induced expression of G2/M phase cell cycle arrest markers. Cells were treated with the indicated concentrations of B220 for 24 h, and cell lysates were analyzed by Western blotting.

Identification of 7-(4'-Cyanophenyl)indoline-1-benzenesulfonamide as a mitotic inhibitor to induce apoptotic cell death and inhibit autophagy in human colorectal cancer cells

Tung-Yun Wu[a], Ting-Yu Cho[b], Chung-Kung Lu[c], Jing-Ping Liou[d], Mei-Chuan Chen[a,b,*]

[a] Ph.D. program for the clinical drug discovery from botanical herbs, College of Pharmacy, Taipei Medical University, Taipei, Taiwan

[b]Graduate Institute of Pharmacognosy, College of Pharmacy, Taipei Medical University, Taipei, Taiwan.

[c]Chinese Medicinal Chemistry, National Research Institute of Chinese Medicine, Taipei, Taiwan

[d]School of Pharmacy, College of Pharmacy, Taipei Medical University, Taipei, Taiwan

***Corresponding author:**

Mei-Chuan Chen, Ph.D. program for the clinical drug discovery from botanical herbs, College of Pharmacy, Taipei Medical University, 250 Wuxing Street, Taipei 11031, Taiwan.

E-mail: mcchen1250@tmu.edu.tw

Uncropped Figures

Fig.2 Wu et al.

C

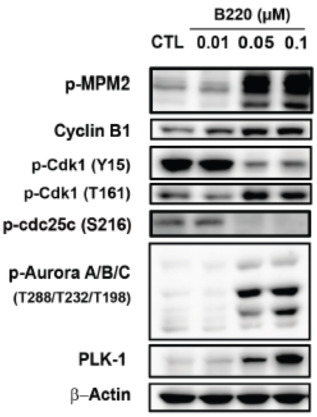

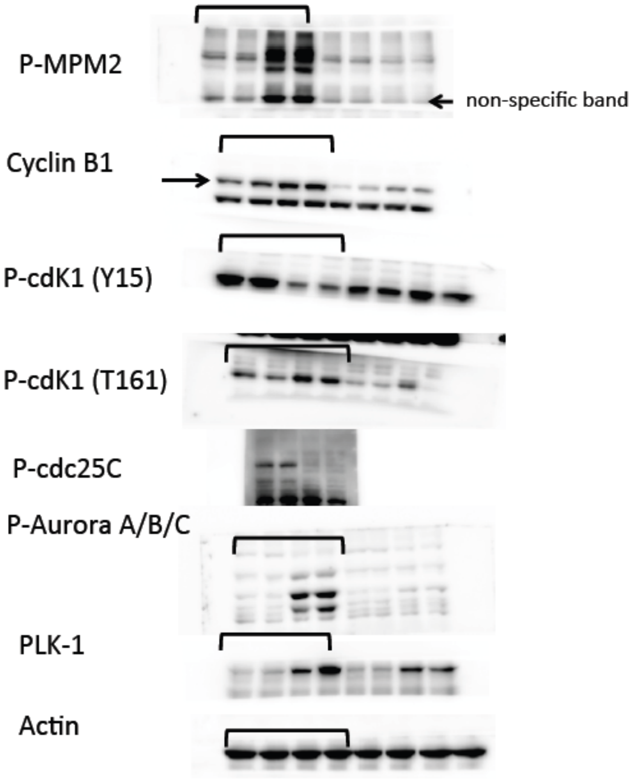

Fig.2 Wu et al.

D

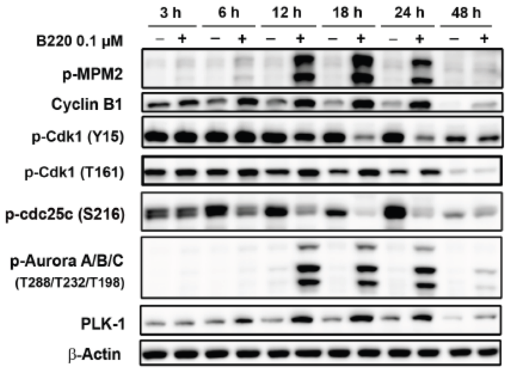

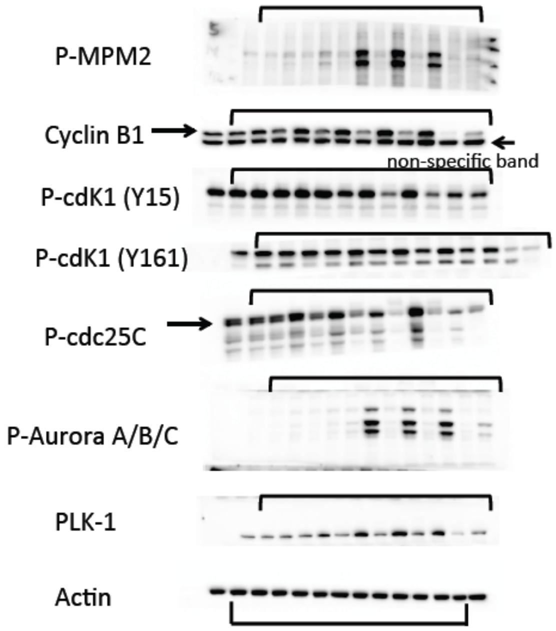

Fig. 3 Wu et al.

A

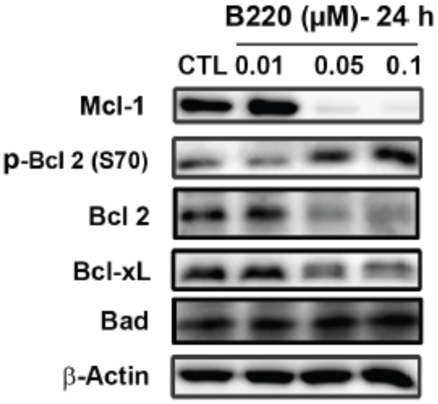

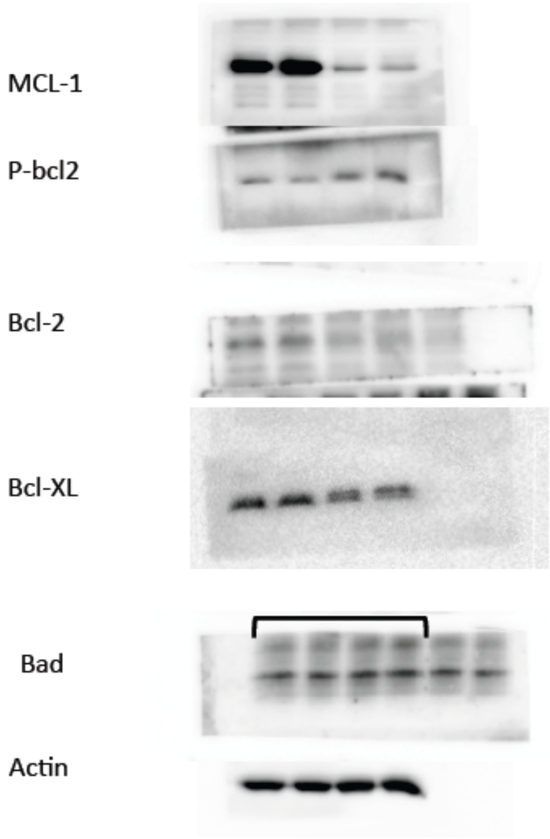

Fig. 3 Wu et al.

B

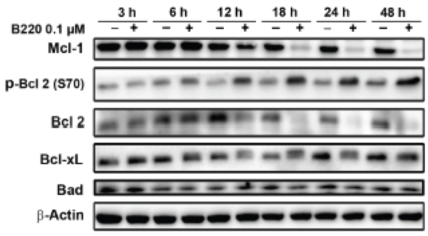

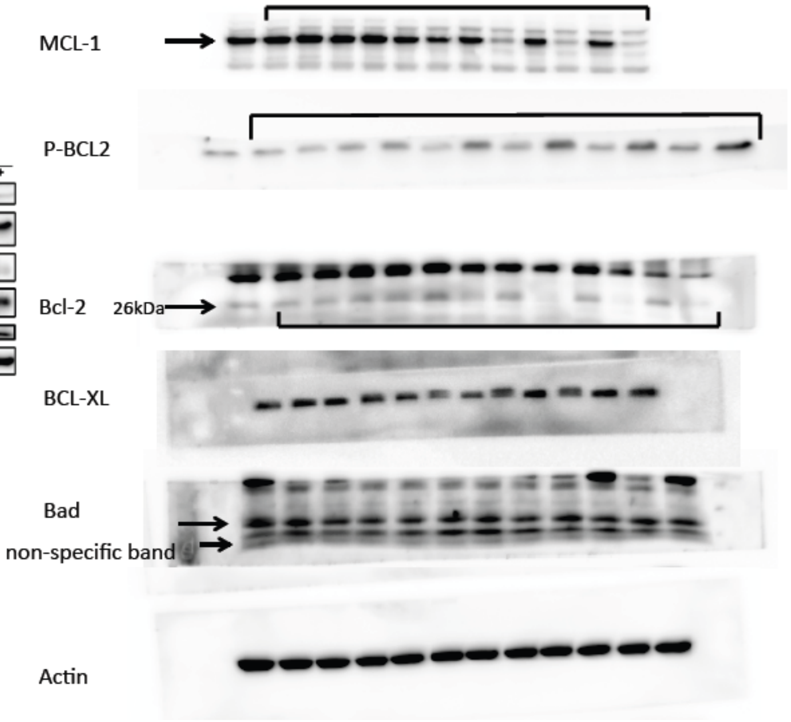

Fig. 3 Wu et al.

C

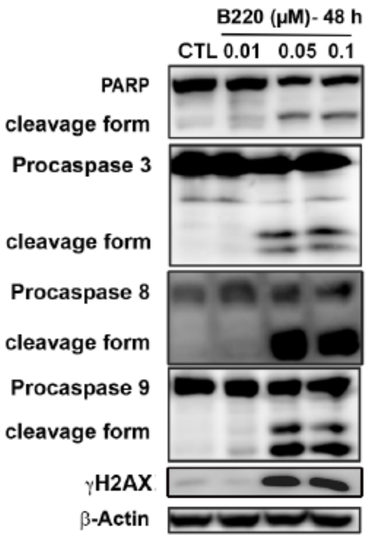

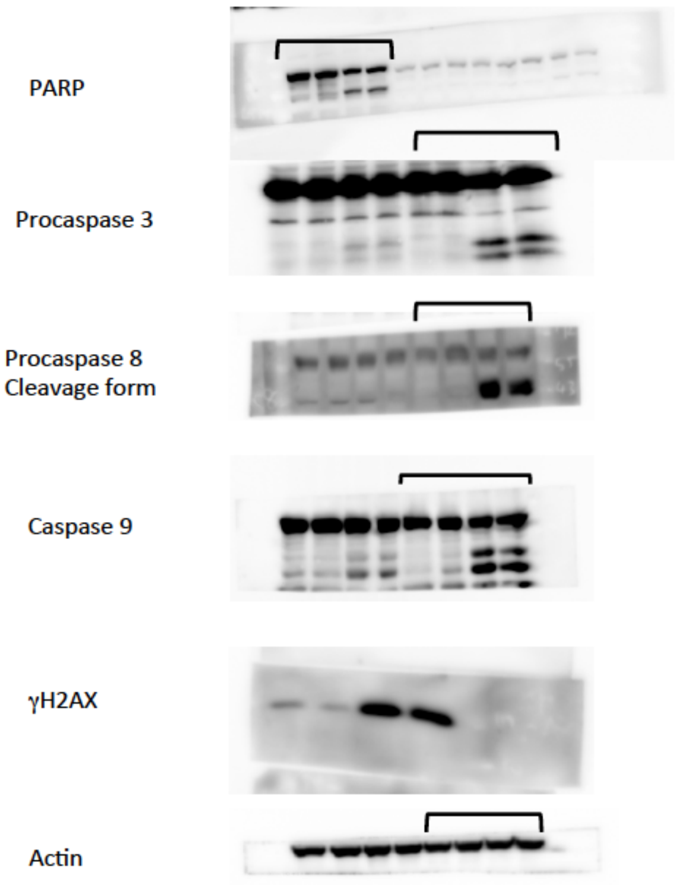

Fig. 3 Wu et al.

D

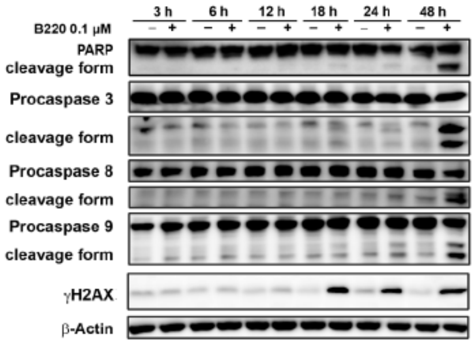

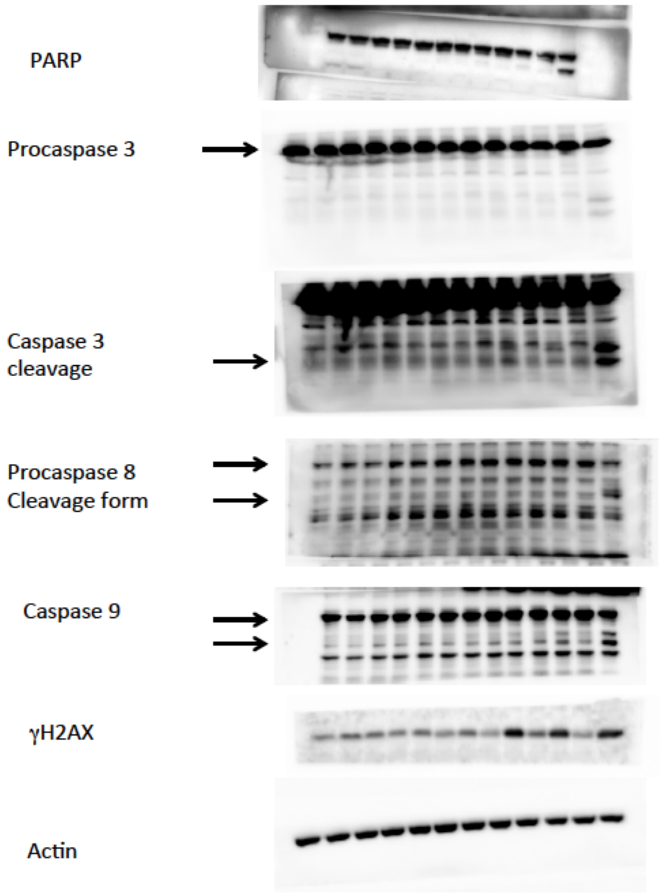

Fig. 4 Wu et al.

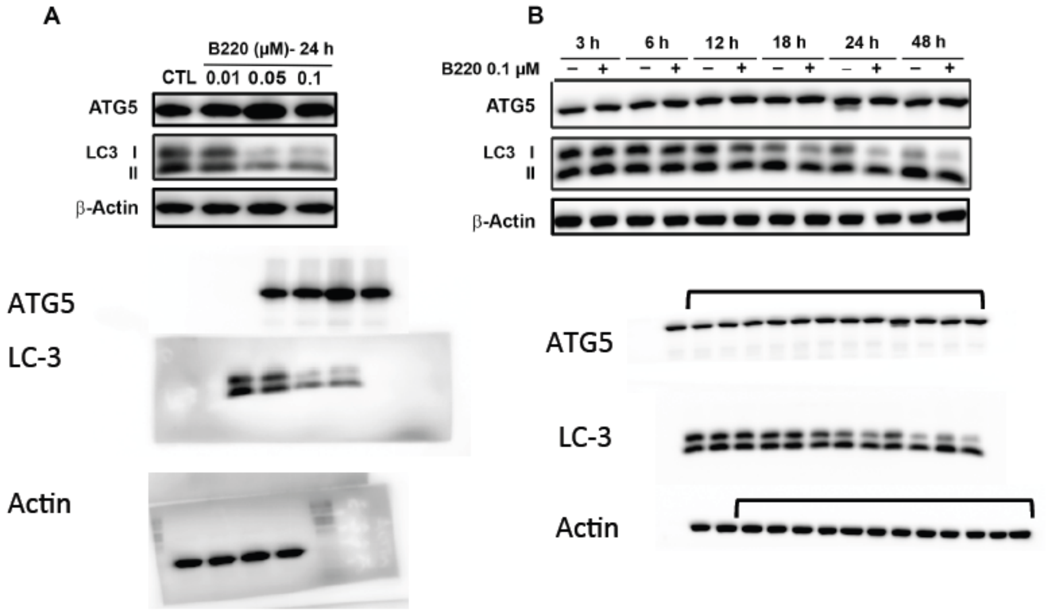

Fig. 4 Wu et al.

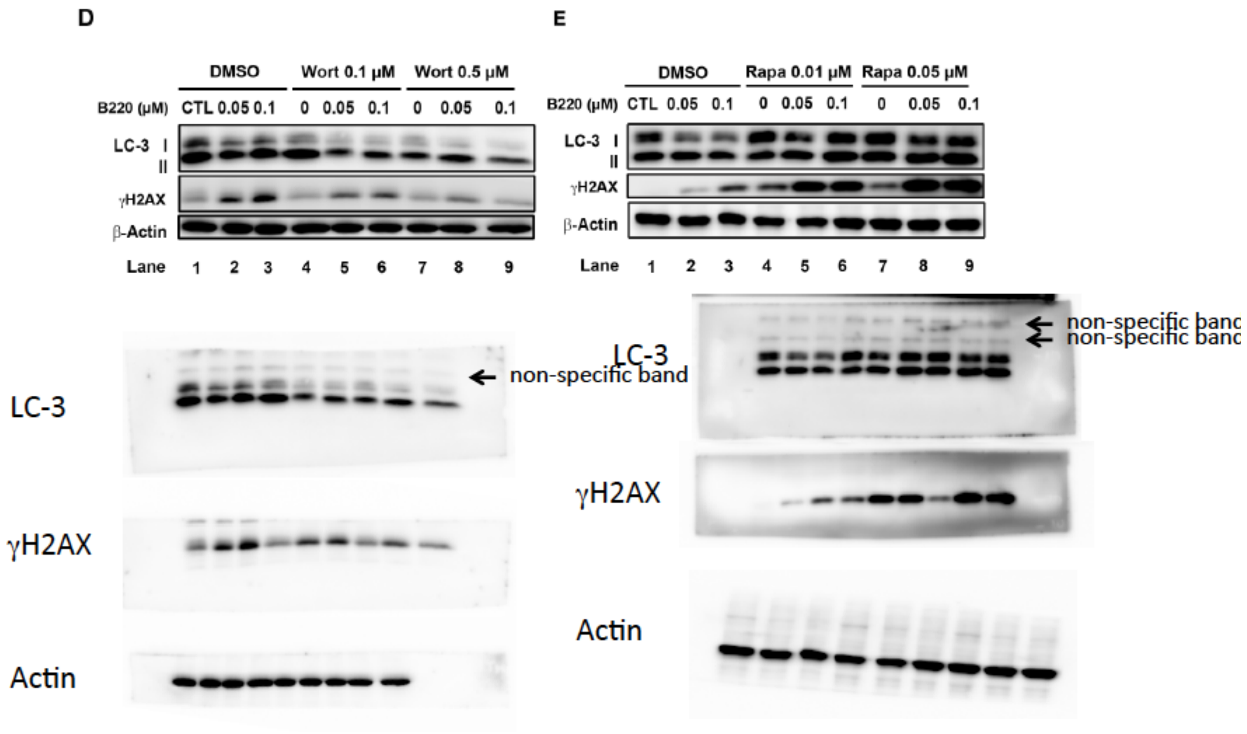

Fig. 5 Wu et al.

A

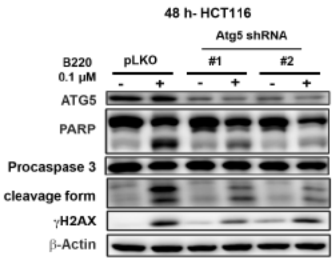

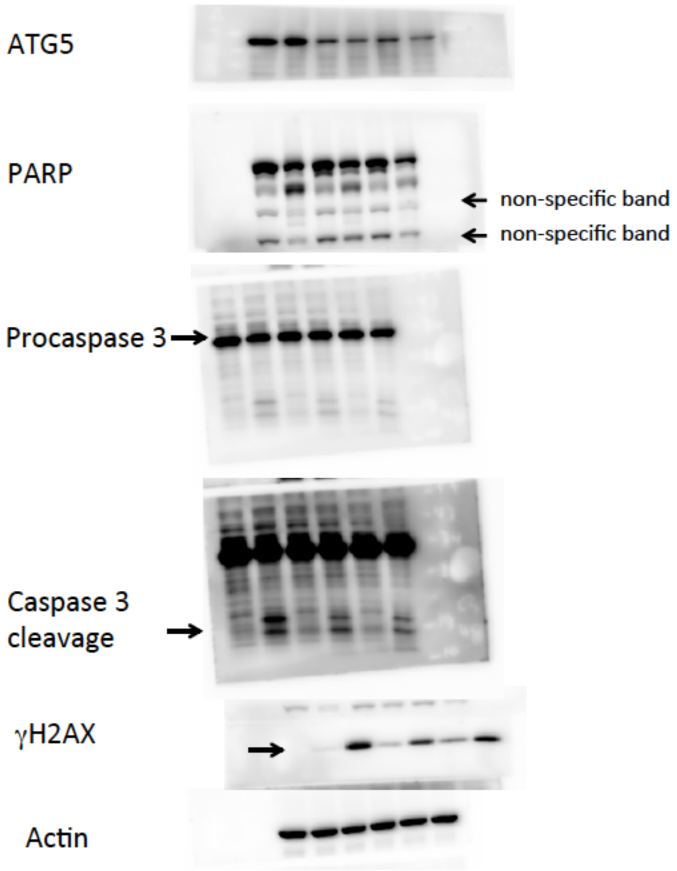

Fig. 5 Wu et al.

C

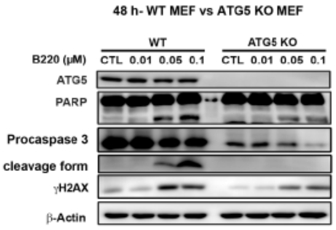

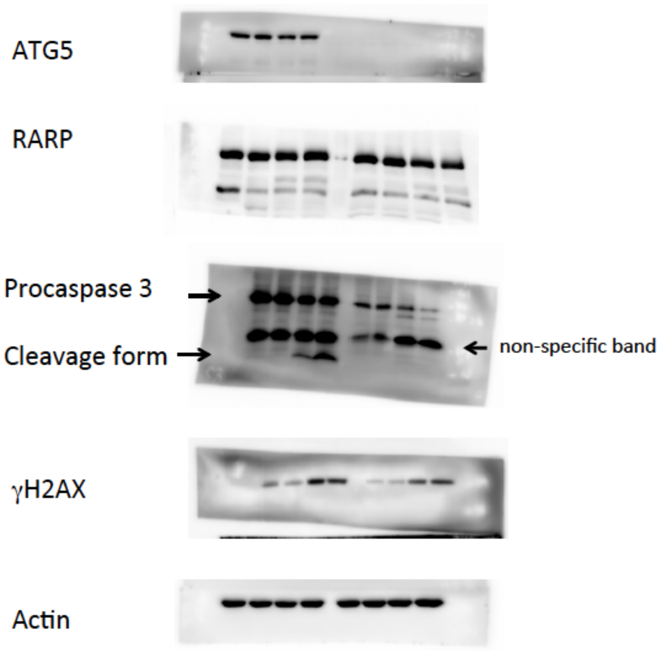

Fig. 6 Wu et al.

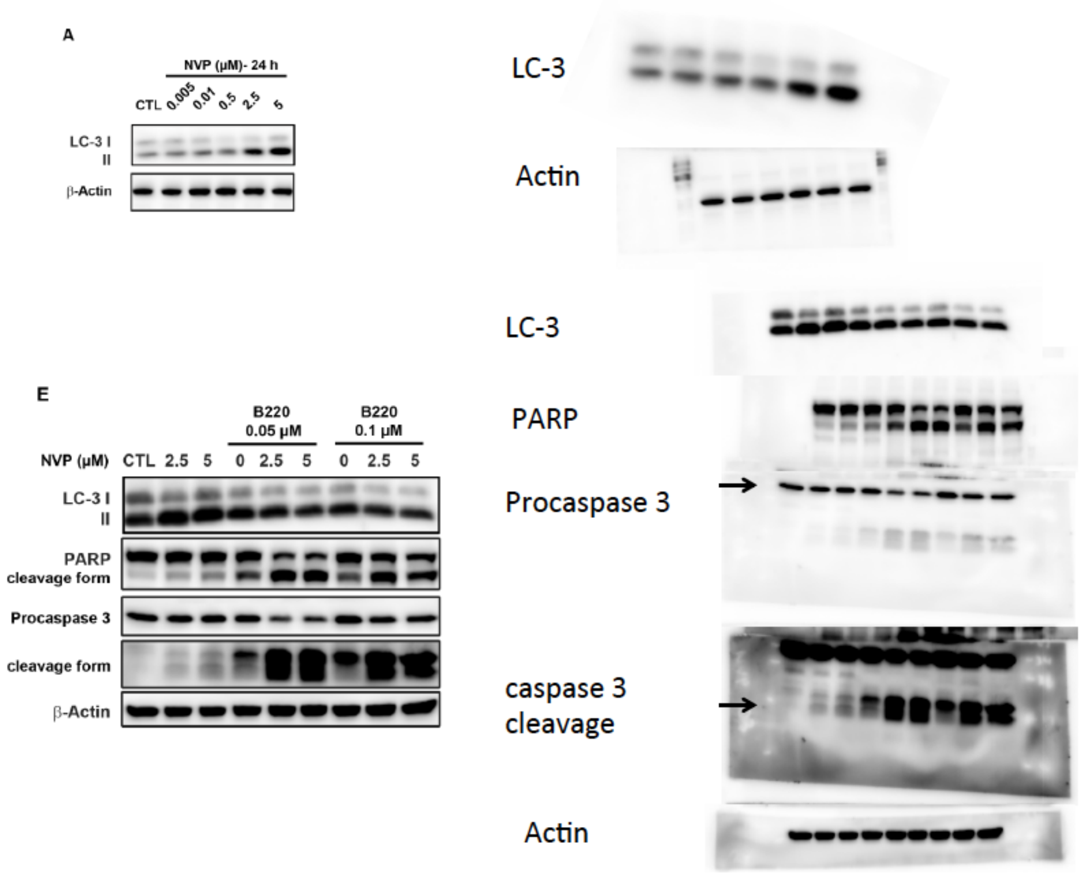
A
NVP (μM)- 24 h
CTL
0.005
0.01
0.5
2.5
5
LC-3 I
II
β-Actin
E
B220
0.05 μM
B220
0.1 μM
NVP (μM)
CTL
2.5
5
0
2.5
5
0
2.5
5
LC-3 I
II
PARP
cleavage form
Procaspase 3
cleavage form
β-Actin
LC-3
Actin
LC-3
PARP
Procaspase 3
caspase 3
cleavage
Actin

Supplementary Fig. 1 Wu et al.

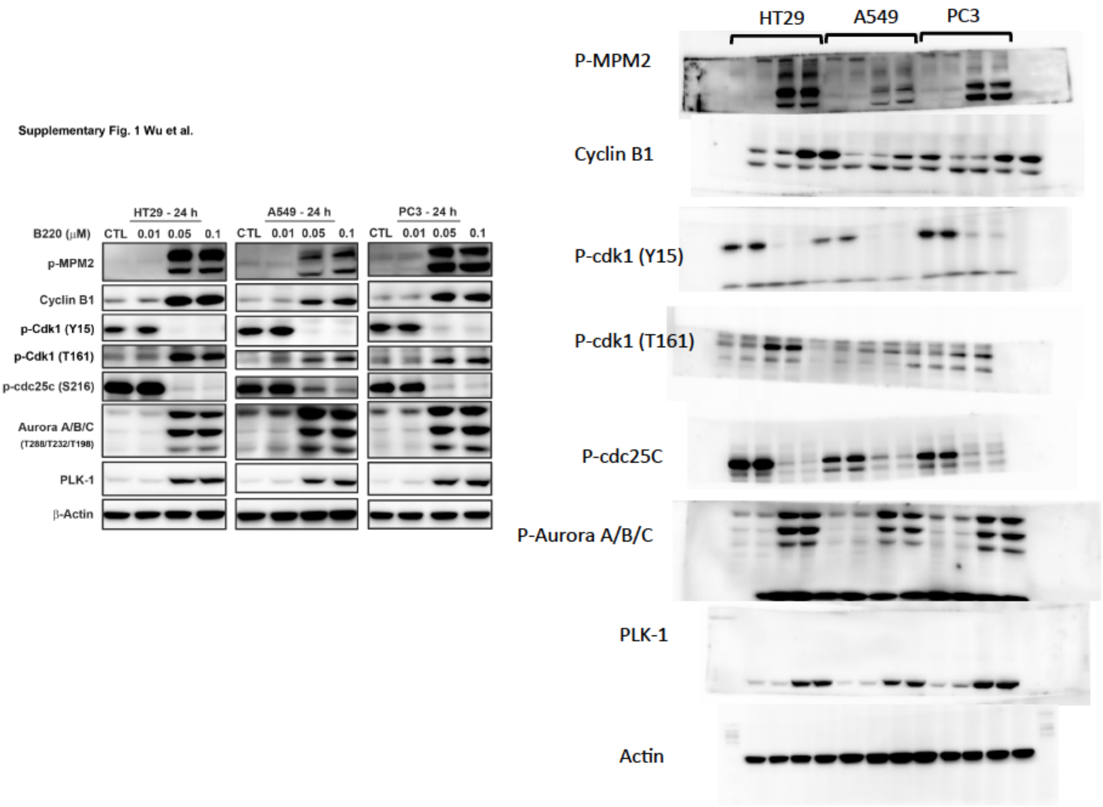

Supplementary Fig. 2 Wu et al.

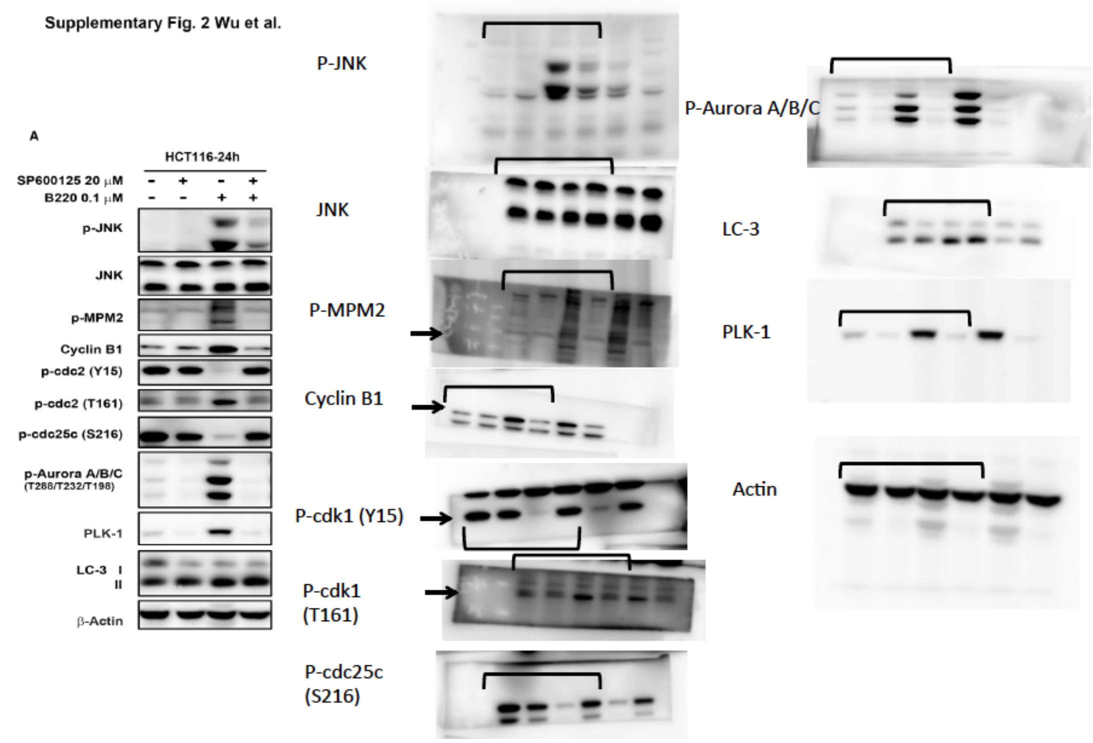

Supplementary Fig. 2 Wu et al.

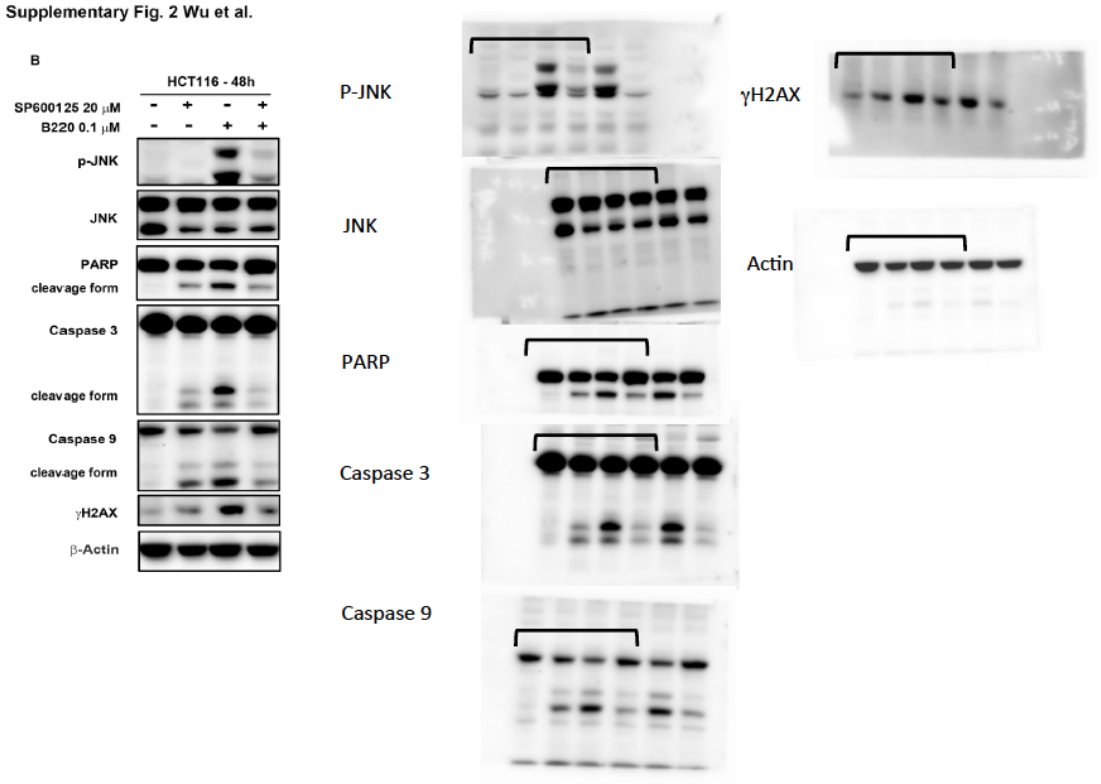

Supplementary Fig. 3 Wu et al.

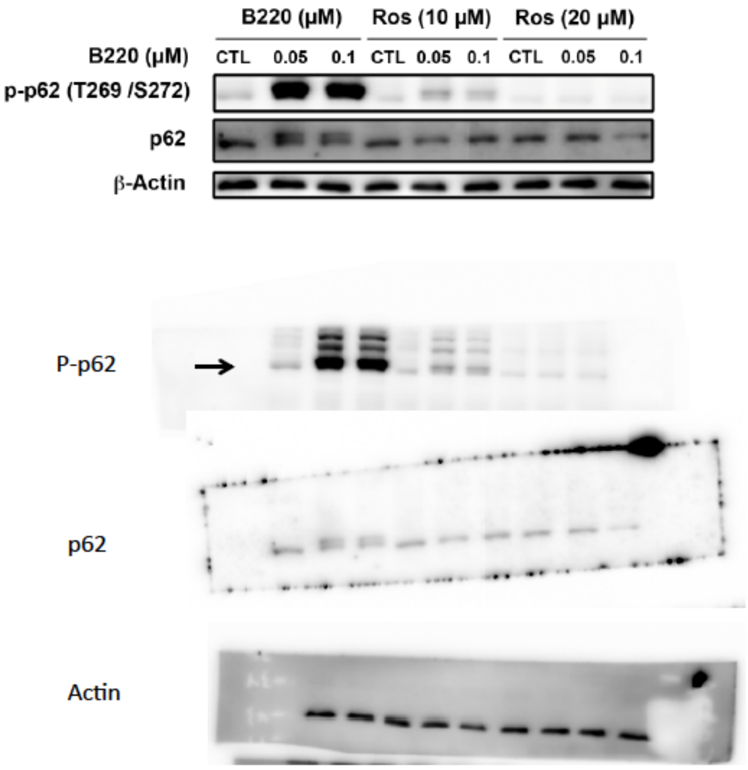

Supplementary Fig. 4 Wu et al.

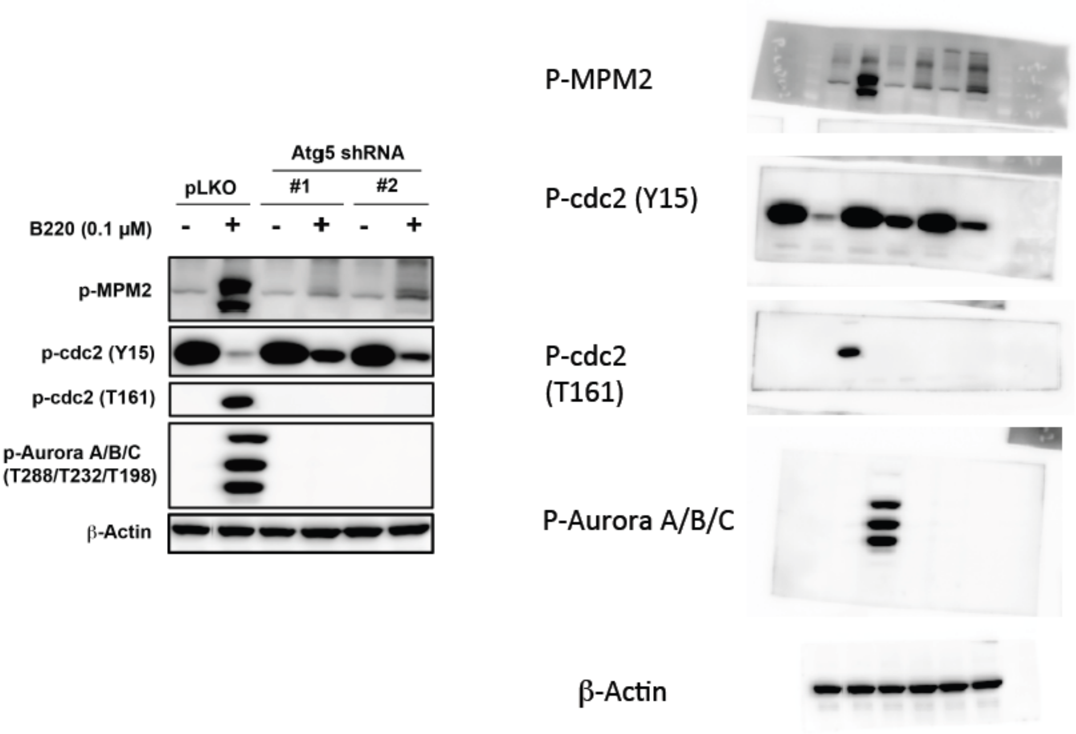

Supplement: Supplementary file 1 — Supplementary Info [file 41598_2017_12795_MOESM1_ESM.pdf]
